# Two-group interlandmark distance analysis for skeletal sex estimation using resampling and posterior probability contour plots

**DOI:** 10.1007/s00414-026-03725-0

**Published:** 2026-02-04

**Authors:** Stefan Schlager, Daniel Franklin, Marco Milella, Andrea Cardini

**Affiliations:** 1 Department of Oral and Maxillofacial Surgery, Medical Center, University of Freiburg, Freiburg im Breisgau, Germany; 2 Centre for Forensic Anthropology, The University of Western Australia, 35 Stirling Highway, Crawley, WA 6009 Australia; 3 Dipartimento di Biologia, Università di Pisa, Via Derna, 1, Pisa, 56126 Italy; 4 Dipartimento di Scienze Chimiche e Geologiche, Università di Modena e Reggio Emilia, Via Campi, Modena, 103 - 41125 Italy

**Keywords:** Bootstrap, Discriminant analysis, Human population, Forensics, Sexual dimorphism, Anthropology

## Abstract

The attribution of taxonomic and/or demographic parameters based on the analysis of interlandmark linear measurements remains a widely used approach in both anthropology and zoology, despite the availability of more sophisticated techniques. Especially in forensic contexts, linear measurements are commonly employed for the estimation of sex and/or ancestry to facilitate identification. In the present study we evaluate the potential of applying a resampling-based approach that uses a newly developed R function to efficiently compute interlandmark distances from their Cartesian coordinates and allows identification of which distances are robustly associated with a biological factor; specifically sex. Following the identification of significant dimorphic measurements, we evaluate their associated sex classification accuracy. Furthermore, we demonstrate how results from a training dataset can be applied to novel cases using a rarely implemented, yet straightforward and effective, graphical method. When limited to two variables, this technique involves generating a scatterplot overlaid with interpolated contour lines representing posterior probabilities. By plotting new observations within this data space, users can visually classify sex and its associated probability, without requirement for further computation, analogous to how elevation is estimated on a topographic map using contour lines. We discuss the advantages and limitations of this novel approach and its statistical reproducibility. Broader application of this method could enhance understanding of population specificity in sexually dimorphic cranial measurements and support the development of contour-based tools for the rapid and accurate estimation of skeletal sex in unidentified human remains.

## Introduction

Alongside the estimation of age at death, population affinity, and stature, estimation of skeletal sex is a cornerstone of biological profile reconstruction in forensic anthropology and osteoarchaeology [1]. In forensic contexts, in particular, accurate sex estimation is essential towards facilitating the identification of unknown individuals—a non-trivial task, given the variable expression of skeletal sexual dimorphism in modern humans and its modulation by genetic, hormonal, and environmental factors [2, 3].

Over the decades this complexity has spurred the development of a plethora of methodological approaches aimed at reliably and accurately capturing size and shape differences between sexes. Morphological methods remain widely used despite the increasing availability of genetic and proteomic techniques [4–7], largely due to their low cost, relative simplicity, rapid data acquisition, reproducibility and acceptable accuracy. These advantages are particularly relevant in contexts with limited resources and strict time constraints. Morphological techniques for the estimation of skeletal sex have been applied to various anatomical regions and include the assessment of both nonmetric morphoscopic traits (the most commonly used approach [8, 9]), linear morphometric measurements [10–12], and geometric morphometric approaches exploring shape variation [13–17].

Group discrimination and correlational analyses based on linear measurements are central to traditional morphometrics [18, 19], with a substantial body of literature supporting applications in estimating both sex and population affinity [20–24]. Traditional morphometric methods have the advantage of being less subjective than morphoscopic approaches and are methodologically simpler than geometric morphometric approaches. By their nature, traditional morphometric approaches emphasize size differences between sexes, particularly in postcranial structures, and to a lesser extent, cranial, mandibular and dental elements [20, 25–27]. Published levels of accuracy in sex classification from such approaches range from a minimum of 70% (cranial base [28]) to a maximum of almost 100% (os coxae [12]), with notable differences between anatomical regions highlighting an overall higher predictive power of postcranial over cranial regions [29, 30]. Further, classification accuracy varies within and between populations, reflective of variances in skeletal morphology “…based on genetic similarities shared from selective and/or environmental influence(s) specific to a defined geographic location” (p19 [31]; and see Franklin & Flavel [32]).

Methodologically, the design of classification models based on linear measurements typically involves three main steps: (1) screening an identified sample to select the measurements with the highest predictive power (e.g., via stepwise discriminant analysis); (2) incorporating the selected measurements into either univariate (e.g., threshold-based) or multivariate (e.g., discriminant function) predictive models; and (3) quantifying the predictive accuracy, either through jack-knife cross-validation and/or using test (hold-out) samples.

Among these steps, the selection of predictive measurements and the evaluation of model accuracy are particularly critical, especially in a forensic context. The screening and selection of measurements become more challenging when dealing with anatomically intricate structures, such as the human cranium, due to a larger number of points and distances to evaluate. The analysis can then become prohibitively demanding if, rather than being limited to a set of traditional craniometric distances, it explores all possible measurements from a defined set of craniometric landmarks (interlandmark distances, henceforth *ILDs*). Since the number of ILDs from the Cartesian coordinates of q landmarks is q × (q – 1)/2, the resulting analyses may indeed involve the evaluation of hundreds, if not thousands, of ILDs. For example, 50 landmarks would correspond to a total of 1225 possible ILDs, many of which will be redundant due to very high correlations between distances measuring similar aspects of size variation. Further, smaller ILDs may lead to an increase in analytical background noise due to higher measurement error in smaller dimensions (Polly, 1998). Thus, prior to any specific use, a careful examination of a large matrix of ILDs, and the parsimonious selection of those that are potentially most useful, is a crucial task.

A second crucial step is the evaluation of the degree of accuracy and applicability of the designed protocols; a task normally approached via cross-validation (i.e., train the model on part of the data and test its classification performance on the withheld portion) and/or external validation on an independent sample. Importantly, overall classification accuracy is evaluated (e.g., pooled-sex or overall classification accuracy) in addition to quantifying correct classification by individual sex. The latter is known as the sex-bias value and quantifies any disproportionate classification of one sex relative to the other. This is important because whilst overall classification accuracy may be high, correct classification by individual sex may be skewed in one direction, rendering the model unsuitable for practical application [33, 34]. The test of new or published methods on samples representing different populations from the training set has a profound biological basis. As noted above, the pattern and magnitude of sexual dimorphism is known to vary across populations due to the combined effect of genetic and environmental factors [32, 35, 36]. Accordingly, sexing methods developed on, for instance, a Central European training set may lead to prediction with low accuracy if blindly applied to forensic casework in Southeast Asia [37]. The growing recognition of population variability as a critical factor in biological profile reconstruction has led to a substantial body of research aimed at testing and refining sex estimation protocols on specific human groups, particularly in forensic contexts [38–41].

In the present study we provide an example of the application of a combination of resampling methods to select, from amongst hundreds of ILDs, a parsimonious subset of measurements highly correlated to an independent two-group factor (sex). More specifically, we examine the stability of squared correlations (Rsq) between ILDs and the factor. Rsq, also known as the coefficient of determination, represents the proportion of variance in a dependent variable that is accounted for by a given predictor within a linear model. It provides a simple and intuitive estimate of the strength of the association between two variables. Thus, focusing on sexual dimorphism in a large sample of adult human crania from a geographically referenced Western Australian population, our resampling approach first employs a newly developed R function [42], called *ILDSR2()* and available in the package *Morpho (≥ 2.13)* [43], to: **(i)** compute all possible pairwise ILDs from the Cartesian coordinates of a configuration of anatomical landmarks; **(ii)** calculate their Rsq to sex and select the ILDs with the highest Rsq; and **(iii)** provide a bootstrap estimate of the strength of the support for the selected sex-related ILDs. In this first part of the analysis, however, instead of directly applying the *ILDSR2()* to the entire dataset, we repeat procedures **i-iii** outlined above, in randomized subsamples. This facilitates further assessment of the impact of sampling error on ILD selection and a more thorough statistical exploration of the results internal validity.

In the second part of the analysis the most dimorphic ILDs are then employed in a linear discriminant analysis (DA) to predict sex in the same dataset (using a leave-one out cross-validation), and in an independent sample from Northern Italy. With the Italian sample, an example of the preliminary external validation of the results from the training dataset is provided. Importantly, the Italian sample demonstrates the effective visualization of shape differences using a graphical approach that, for brevity, is termed the posterior probability (PP) contour plot. Whilst the latter approach appears to be rarely applied in a forensic context, it certainly holds significant potential to be important part of the toolkit deployed in a routine anthropological assessment; with only two measurements, this type of data scatterplot, with contour lines estimating probabilities of being male or female, provides a very effective and simple graphical tool for the rapid estimation of skeletal sex.

## Materials and methods

### Data

**Fig. 1:**
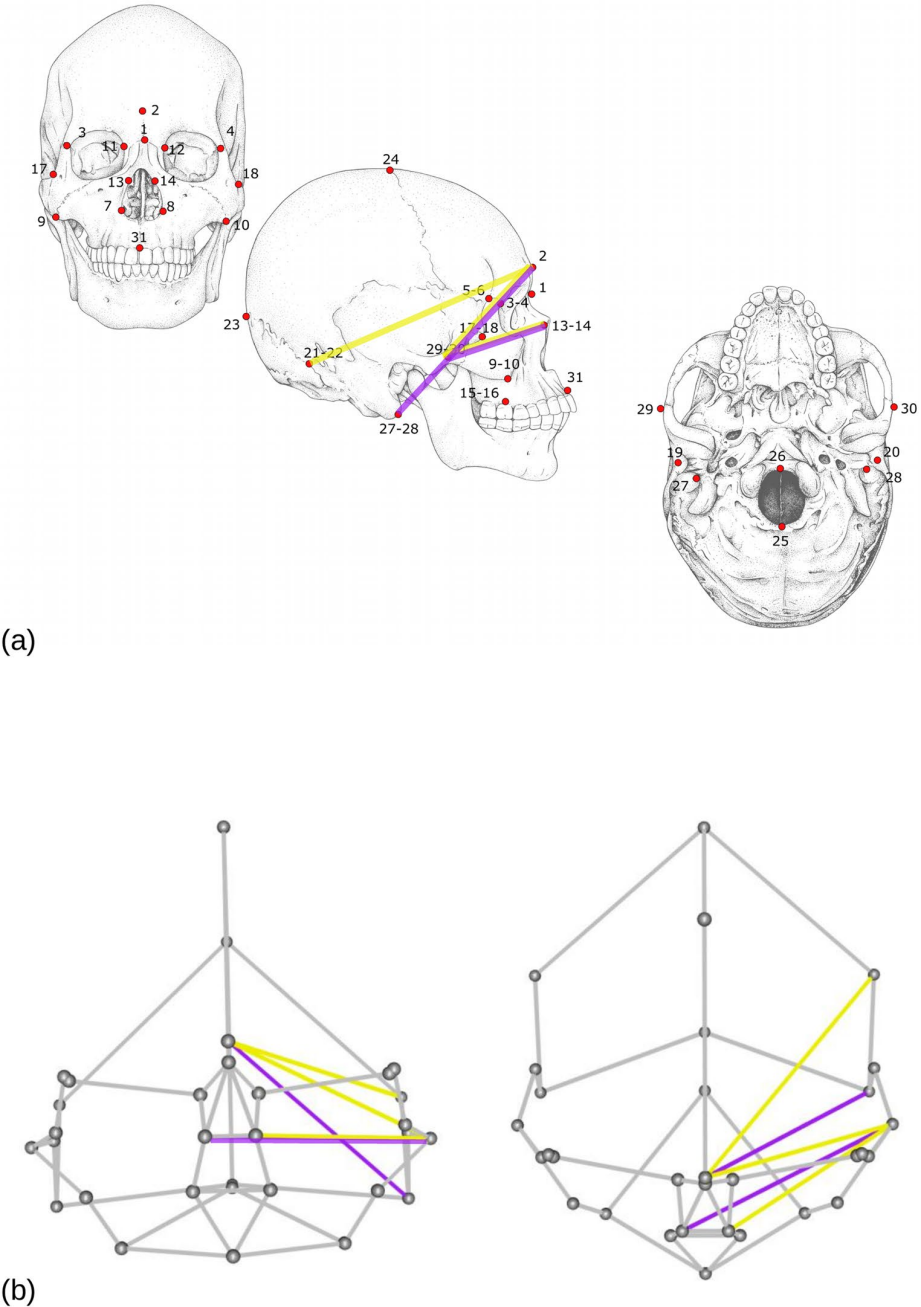
(**a**) Landmark configuration using abbreviations for the full names provided in Table 1 and links for best supported top sex-related ILDs (violet, support > 50%; yellow, support > 10% but < 50%; see Results and Fig. 2). (**b**) Wireframe diagram, seen frontally (left) and from above (right), showing a stylized cranium obtained by linking pairs of landmarks; best supported top sex-related ILDs shown as in (**a**)

The training sample comprises 100 female and 100 male individuals from a contemporary Western Australian population. Previous research on the same dataset using geometric and traditional morphometric cranial analyses demonstrated that sex can be estimated with between 84 and 88% accuracy [15]. From the original data, however, a reduced configuration of 31 3D landmarks are selected (Fig. 1). With a few approximations, these landmarks are shared with another study of cranial sexual dimorphism, based on the analysis of archaeological remains from Bologna, Northern Italy. That sample comprises 122 adults, of whom 56 are female and 66 male, with dates of death between the 19th and early 20th centuries [44]. Thus, both previous analyses examined the predictive power of cranial morphology to classify sex in adult humans. However, while Franklin and colleagues used CT data from living individuals, Milella and coauthors measured landmarks directly in physical crania using a 3D digitizer. The fact that the data were collected with different instruments by different operators is an important issue, but one of secondary relevance in the context of our study. This is because the Italian sample is only employed to provide a proof of concept in how to explore the external validity of the DA of the Australian dataset when applied to an independent sample. Readers interested in further details on the two datasets can refer to our previous papers [15, 44].

**Table 1:** Landmarks configuration using landmarks in common between Franklin et al. [15] (FRA) and Milella et al. [43] (MIL). The corresponding landmark names used in the two studies are shown next to the number of the landmark used by the authors

This study	Side	FRA		MIL	
#		#	Name	#	Name
1	midplane	1	nasion	1	nasion
2	midplane	2	glabella	54	glabella
3	right	6	frontozygomaticorbitale	21	frontozygomaticorbitale
4	left	11	frontozygomaticorbitale	20	frontozygomaticorbitale
5	right	7	frontomalare anterior	23	~ frontomalare temporale
6	left	12	frontomalare anterior	22	~ frontomalare temporale
7	right	13	alare	41	alare
8	left	14	alare	40	alare
9	right	17	zygomaxillare anterior	49	zygomaxillare
10	left	18	zygomaxillare anterior	48	zygomaxillare
11	right	19	dacryon	33	dacryon
12	left	33	dacryon	32	dacryon
13	right	20	nasomaxillary	37	nasomaxillary
14	left	34	nasomaxillary	36	nasomaxillary
15	right	21	ectomolare	47	ectomolare
16	left	32	ectomolare	46	ectomolare
17	right	22	jugalia	25	jugale
18	left	30	jugalia	24	jugale
19	right	24	porion	61	porion
20	left	31	porion	60	porion
21	right	25	asterion	59	asterion
22	left	28	asterion	58	asterion
23	midplane	26	lambda	50	lambda
24	midplane	27	bregma	52	bregma
25	midplane	35	opisthion	81	opisthion
26	midplane	36	basion	82	basion
27	right	39	mastoidale	69	mastoidale
28	left	40	mastoidale	68	mastoidale
29	right	41	zygion	65 − 13	~ zygion
30	left	42	zygion	64 − 12	~ zygion
31	midplane	16	prosthion	57	~ alveolare

*Several FRA landmarks were approximated in the MIL dataset. Specifically, the MIL frontomalare temporale was used as a proxy for the FRA frontomalare anterior, although it is positioned slightly more posteriorly. The FRA zygion was estimated as the average location of the MIL superior zygotemporale and the articular eminence, while the FRA prosthion was approximated using the MIL alveolare. Despite these approximations, none of the substituted landmarks exhibited unusually high variance in form space, as assessed following Procrustes superimposition and rescaling of the configurations to their original size using centroid size

For convenience, the data are imported as Cartesian coordinates of 3D landmarks in a form space obtained by Procrustes superimposing without scaling [45]. During this operation, small asymmetries were discarded by selecting only the symmetric component [46]. When ILDs are analysed this is equivalent to averaging corresponding ILDs, which measure the same bilateral interlandmark cranial measurements, and are, therefore, mostly redundant. Neither using form or symmetrizing the data is a required step, however. We could have used the raw landmark coordinates, as measured originally, including the asymmetric component. If asymmetries are discarded, one should bear in mind that the measurements contain duplicated data and part of the results also include duplicates that must be removed. For instance, in symmetrized data, alare to zygion measured on the right side is identical to alare-zygion on the left side. If one of the two landmarks is on one side and the second landmark on the opposite one, however, we get a different pair of identical ILDs, both crossing the midplane. In these two examples of redundant ILDs, only one measurement in each pair is informative. The other one can be ignored or excluded. Together with the 31 3D landmark coordinates, we also imported the sex factor coding females as F and males as M, an abbreviation henceforth applied throughout this article.

### ILDs calculation and resampling approach to assess data support for sex-related ILDs

A description of *ILDSR2()*, all its arguments and the values it returns, is available in the help of the R *Morpho (≥ 2.13)* package [43] by running the command *?ILDSR2*. Here, we focus only on the specific settings used in our study case. However, we stress that the factor, whose association with ILDs is explored using the function, can be a grouping variable with two levels (as in our example) but it could also be an ordinal or continuous covariate (e.g., age or another morphometric descriptor, such as body mass or height). The landmark coordinates of the Australian sample were loaded in the function as a 3D array, a customary format for this type of data in R [47]. Sex, a two-level factor, was the group variable. Next we specified R2tol, which is the upper percentile for the Rsq. In our dataset with 31 landmarks, there are 465 ILDs, which were used, one by one, to calculate their Rsq in relation to sex. Using R2tol = 0.98, we selected the ‘top-ten’ ILDs with highest Rsq (i.e., the top 2% of all 465 Rsq, which we refer to as ‘top ILDs’). In fact, however, the real number of top ILDs can be as low as just five, if all the top ILDs happen to be symmetric and half of them is, therefore, redundant, as previously explained. The R2tol is arbitrary. It should be finely-tuned to the specific data and aim. As our aim is forensic and for practical applications we want a small number of simple predictors of sex, we used a fairly restrictive R2tol.

After running the function, *ILDSR2()* can output the matrix of all 465 ILDs, as well as the corresponding sex-related Rsq, for further analyses. If we call the results in R *res*, ILDs and Rsq, in descending order from the highest to the lowest, are obtained with the commands *res$sampleILD* and *res$allR2* respectively. Up to this point, computations are trivial and can be done in a number of other programs, including a free and user-friendly software such as PAST [48]. However, *ILDSR2()* also allows to explore how strongly the available data support the Rsq of the top ILDs by bootstrapping. In the two factor case, the individual groups are resampled with replacement, thus adding within group noise regarding group means and downstream results. For instance, in a sample of ten individuals labelled with ordinal numbers from 1 to 10, the original sample is “1, 2, 3, 4, 5, 6, 7, 8, 9, 10”, whereas a bootstrapped pseudo-sample could be “1, 1, 3, 4, 5, 5, 5, 7, 9, 10”, thus having the same sample size but missing some observations and including others multiple times. The same analyses performed on the original sample are then applied to the bootstrapped pseudo-samples. When this resampling procedure is repeated many times, it allows the signal in the original data to be evaluated against a background of noise by simulating uncertainty due to sampling error, assuming that the original dataset is reasonably representative. In the regression case, where the ILDs are evaluated in respect to a covariate, all data (landmark data and covariate) are resampled with replacement to obtain the backdrop noise against which the original signal is to be compared. This is useful because, especially in small samples, even the largest Rsq could in fact be poorly supported by the data. To specify this option, we used the argument *wg.rounds* = 99 that asks the function to separately bootstrap the sample of each group (females and males, in our case) 99 times and recompute the top ILDs Rsq in the corresponding pseudosamples. The function keeps track of how many times an observed top ILD ends up again as a top ILD, which in our case meant the top 2% Rsq. This number is divided by the total number of replicate analyses to provide the proportion (or percentage, if multiplied by 100) of bootstrap support for a top ILD. Using 99 bootstraps makes the denominator equal to 100 when results are summarized (likewise, with 999, the denominator would be 1000 etc.). This is convenient to obtain round numbers, because the observed result (i.e., the analysis of the original sample) is included in the counts both at the numerator and denominator. We considered a top ILD Rsq well supported when found at least in 50% of the bootstrapped samples. As with the number of randomized subsamples (next paragraph), a 50% threshold seems appropriate to estimate support in our analysis. One could choose a more stringent criterion (e.g., 90%) but then incur the risk of finding almost no ILD supported by the data.

As anticipated in the Introduction, to further explore how sample-dependent results are, instead of applying *ILDSR2()* to the total sample of 200 individuals, we used the function in randomized subsamples of 50 women and 50 men. The randomized subsamples were obtained with an ad hoc script in R. This randomized subsampling experiment was repeated 100 times. Our expectation was that some top ILDs might be less dependent than others on the specific composition of the subsamples. The randomization is not the same as simulating 100 separate samples, because randomized subsamples are not mutually exclusive and, thus, truly independent. For example, some individuals present in the first randomized subsample will be present also in the second one, and so on. Nonetheless, in relation to the available data, the analysis allows to exclude top ILDs that are more strongly biased by the precise composition of the sample.

### Discriminant analysis using best supported top ILDs and posterior probability contour scatterplot

The top ILDs, whose high Rsq with sex was supported by 50% of the bootstraps in at least 50% of the randomized subsamples, were selected as the set of linear cranial measurements for predicting sex in a leave-one out cross-validated DA. The DA was performed using the *Morpho* function *CVA()*. Because there were only two predictors (see Results), to illustrate group separation, we employed a simple scatterplot of the Australian sample in the data space of the two selected top ILDs, instead of plotting individuals on the vector of DA coefficients, as conventional [49]. In the scatterplot, we colour-coded the symbols (circles for females and squares for males) with a pink to blue gradient of tones proportional to the DA posterior probability (PP) of males. Thus, with a PP of 1, an individual was coloured in dark blue and classified as a male with 100% confidence. With a male posterior probability of 0, an individual was coloured in bright pink and predicted to be female with 100% confidence. Intermediate tones implied intermediate levels of confidence in the classification. In the scatterplot, we added thin-plate spline contour lines to approximate the boundaries of the PPs in the ILD data space at 0.1 intervals. Thus, contour lines in the plot estimated PPs of 0.0, 0.1, 0.2 … 0.8, 0.9, 1.0. The thin plate spline interpolation was computed using the default options of the function *Tps()* of the package *fields* [50]. Using the real data space, there is no issue with overfitting, which may happen when projecting observations on a DA vector of coefficient [51]. Moreover, the top ILD scatterplot can be used to predict the sex of a new individual (not included in the DA) by plotting their data onto the PP contour plot. This approach is analogous to locating a geographical point using latitude and longitude and estimating its elevation using equal altitude contour lines. In this context, the individual’s approximate PP can be inferred based on their position relative to the boundaries of the PP contour plot. To demonstrate this method of graphical prediction, we utilized data from Milella et al. [44] to calculate the same two ILDs that were identified as the most informative for sex discrimination in the analysis of the Australian sample. These observations were subsequently added to the Australian sample’s PP contour plot using green filled circles for Italian females and orange filled squares for Italian males.

## Results

Depending on the randomized subsample, and after retaining only one identical symmetric ILD, we consistently identified between one and four non-redundant top ILDs, each with a bootstrap support of at least 50%. In total, the 100 randomized subsamples produced 23 different top ILDs, which represent approximately 9% of all possible non-redundant ILDs using 31 landmarks. Thus, in spite of partially overlapping samples, which inevitably include a random number of the same individuals, ILDs with the highest best supported Rsq for sex showed a fair amount of variability depending on the precise composition of a sample.

**Fig. 2:**
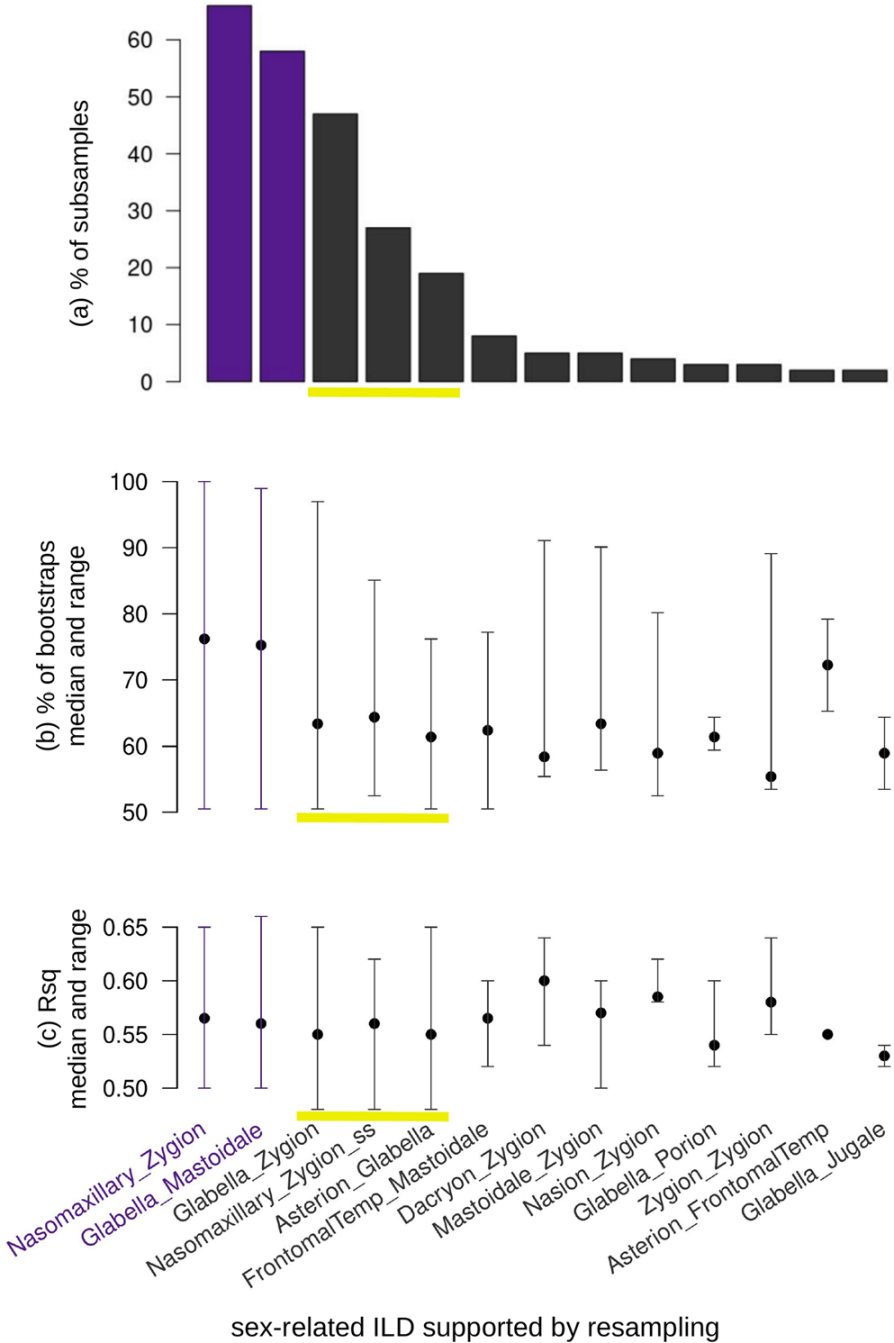
Summary of resampling analyses using the Australian dataset: (**a**) percentage of randomized subsamples (only shown when above 2%) supporting each top ILD, listed from highest to lowest; median, minimum, and maximum values for (**b**) the percentage of bootstrap support for top ILDs and (**c**) their Rsq values across the randomized subsamples, whose bootstrap support was above 50%. For instance, NAZYG was a well supported (> 50% of bootstraps) top ILD in 64 of the random subsamples (Fig. 2a), with support (Fig. 2b) ranging between 51% and 100% (median 76%); in contrast, glabella-jugale, at the opposite end of the plot (Fig. 2a-b), was supported as top ILD by more than 50% of bootstraps only in two randomized subsamples (Fig. 2a), once with a bootstrap support of 54% and the other time of 64%, yielding a median of 59%, exactly halfway between those two values (Fig. 2b). Likewise, in Fig. 2c, the median, minimum, and maximum Rsq values for NAZYG summarize the Rsq values of those 64 subsamples, whereas for glabella-jugale they correspond to the two Rsq values from the only randomized subsamples in which this measurement was well supported as a top ILD. To better stress the most promising measurements for sex discrimination, results of the ILDs supported in more than 50% of randomized subsamples are emphasized in violet, whereas a yellow underscore is used for those below 50% but above 10% support. ILD names are shown at the bottom of the figure. For ILDs involving two bilateral landmarks, if the suffix “ss” is added (which happens only once), it means that the ILD had both landmarks on the same side (e.g., the left); if the suffix is absent, one landmark is on one side (e.g., left nasomaxillary) and the other on the opposite one (e.g., right zygion); the specific side is irrelevant, since crania were symmetrized

In Fig. 2, that summarizes the main findings from the resampling experiments, we omitted the top ILDs supported by only a single randomized subsample and, among the remaining 13 top ILDs, we highlighted in violet the only two supported by more than 50% of the randomizations. These were glabella–mastoidale and, with the two landmarks on opposite cranial sides (i.e., one on the left and the other on the right or vice versa) nasomaxillary–zygion. For conciseness, we will hereafter abbreviate their names as GLAMA and NAZYG, respectively. Although only these two ILDs were eventually used in the DA, three others (highlighted with a yellow underline in Fig. 2) appeared in more than 10% (but less than 50%) of the randomized subsamples, and will be briefly discussed later.

**Fig. 3:**
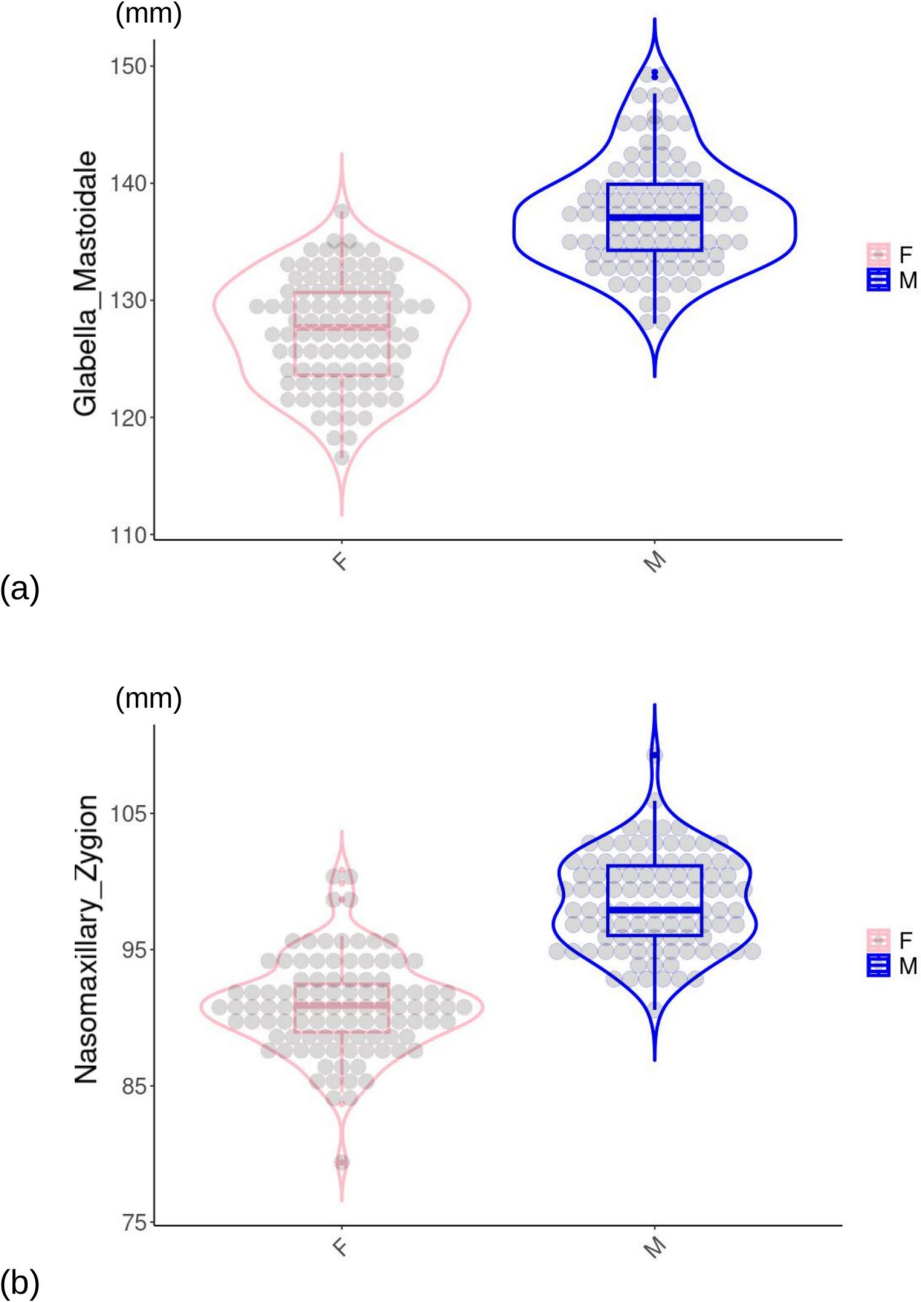
Box and jitter violin plots for the best supported sex-correlated top ILDs: (**a**) GLAMA; (**b**) NAZYG

Individual variation of GLAMA and NAZYG in the total sample of 200 adults is summarized with box and violin plots (Hintze & Nelson, 1998; Wickham, 2011) for each sex in Fig. 3. The plots confirmed the good separation of women and men using GLAMA and NAZYG, as in both instances the upper margin of the box for the females was consistently well below the lower margin of the box for the males. However, depending on the subsample, the bootstrap support of these two ILDs varied approximately from 51% (the minimum to be counted as a well supported top ILD in a specific subsample) to almost 100%, with an average of ~ 75% (Fig. 2b). The Rsq of bootstrap-supported GLAMA and NAZYG also varied remarkably across subsamples from approximately 0.50 to 0.65 (Fig. 2c). A third ILD, glabella-zygion, which was just below the 50% threshold for the randomized subsamples (Fig. 2a), also showed a fairly large variation in the percentages of bootstrap support (Fig. 2b), as well as in the corresponding Rsq (Fig. 2c). The average bootstrap support of asterion-frontomalare temporale (Fig. 2b), as well as its Rsq (Fig. 2c), was almost as high as for GLAMA and NAZYG, but those values originated from the only two randomized subsamples in which asterion-frontomalare temporale was a top ILD in more than 50% of bootstraps. Likewise, a few other top ILDs (e.g., dacryon-zygion, nasion-zygion and zygion-zygion) with an average Rsq larger than GLAMA and NAZYG were all well below the 50% support threshold in the randomized subsamples. Thus, as with asterion-frontomalare temporale, those large Rsq also originated from a small number of subsamples and are, therefore, weakly supported.

**Fig. 4:**
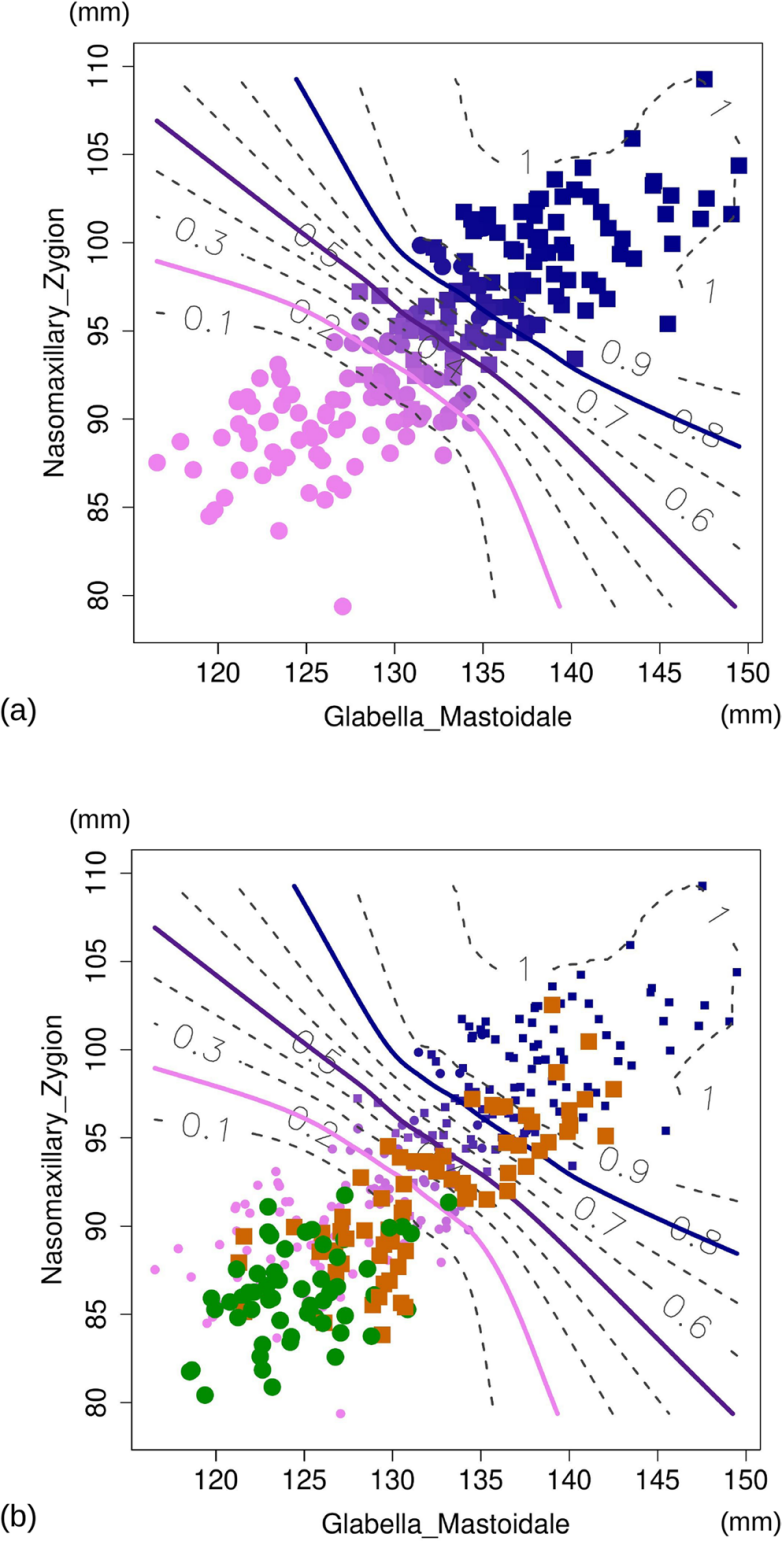
PP contour scatterplot (**a**) of GLAMA-NAZYG using all 200 individuals. DA PPs of males are visualized, as explained in the methods, using a colour gradient from dark blue to bright pink (filled symbols using circles for females and squares for males). Observed PPs are interpolated using a thin-plate spline contour plot to estimate male PP of 0.1 (i.e., 0.9 for females), 0.2 (i.e., 0.8 for females) etc. in the GLAMA-NAZYG data space. The midpoint posterior probability (0.5) used to classify individuals as male (> 0.5) or female (< 0.5) is shown in violet. Pink and blue highlight posterior probabilities of 0.2 and 0.8, representing 80% confidence boundaries for females and males, respectively. In (**b**), individuals from the Italian sample of Milella et al. [43] are projected onto (**a**), with green filled circles for females and orange filled squares for males

The DA using GLAMA and NAZYG to predict sex in the total sample of 200 individuals had an average cross-validated accuracy of 90% with a slight (+ 5%) female bias. (Table 2). Figure 4a shows the scatterplot of the Australian sample in the data space of GLAMA and NAZYG. In this figure, as explained in the Methods, individuals are colour-coded according to male PP with contour lines showing interpolated male PP boundaries. Even if contour lines represent the estimated PP of males, corresponding lines for females would be identical, as a female PP is equal to one minus the male PP.

**Table 2:** Classification accuracy in a leave-one out cross-validated DA using GLAMA and NAZYG

Validation	Sex	F	M*	Average	F-biased
LOO-ILDs	F	92	8		
	M	13	87	90	5

*One unusually large male individual is correctly classified as male with highest posterior probability but a typicality probability of less than 0.01

Overlap between females and males was minimal in the scatterplot and most individuals were affiliated to the correct group with a high PP (75% correct classification in both sexes with a PP ≥ 0.8 for that sex). Nonetheless, one fourth of females and males occupied the central ‘low confidence’ (sex-specific PP < 0.8) region of the GLAMA and NAZYG data space, where there was some overlap between females and males. A minority of individuals (10%) crossed the 0.5 boundary, thus being misclassified (i.e., a woman identified as a man or vice versa).

After calculating the GLAMA and NAZYG ILDs for the Italian sample, we plotted them in Fig. 4b within the data space of the Australian dataset. Because both measurements tended to be shorter in the Italian sample, the data points were generally located closer to the lower left corner of the plot. With Italian women being on average smaller than Australian ones, they were all classified as females with high confidence except one, whose PP was slightly below 0.8 for females. In contrast, because of the smaller size of Italian males, they partly overlapped the range of variation of the Australian females, and most were thus either misidentified as females or classified as males with low (< 0.8) PP.

## Discussion

### Limitations of the new R function and optional plots

In the present study we demonstrated how to apply *ILDSR2()* to Cartesian coordinates of anatomical landmarks in order to compute linear distances and explore their correlation to a two-group factor, such as sex. Data can be either 2D (measured on flat images) or 3D landmarks (as in our case). With 2D data, however, the flattening of the third dimension may introduce a distortion that makes ILDs inaccurate [52, 53]. Currently the function is limited to univariate correlations with a two-group factor or a single covariate, but future versions may include more options and support for more complex analyses. This would be useful, for instance, in research on population differences, that usually include several geographic samples in the analysis, as well as in correlational analyses with multiple covariates. For now, users can modify the function themselves for specific uses, do pairwise group comparisons or, for multiple covariates, employ the first principal component of a set of covariate as a first, albeit potentially inaccurate, approximation.

Together with the main analytical results (top ILDs Rsq and bootstrap percentages), *ILDSR2()* produces a number of graphical summaries we have not used in our example. As we explain below, we describe these plots here because they are optional and appropriate only in a special case, which is when landmark data are in a specific form space. Thus, as we illustrate the graphics, we discuss what information they might provide and when they can be used.

**Fig. 5:**
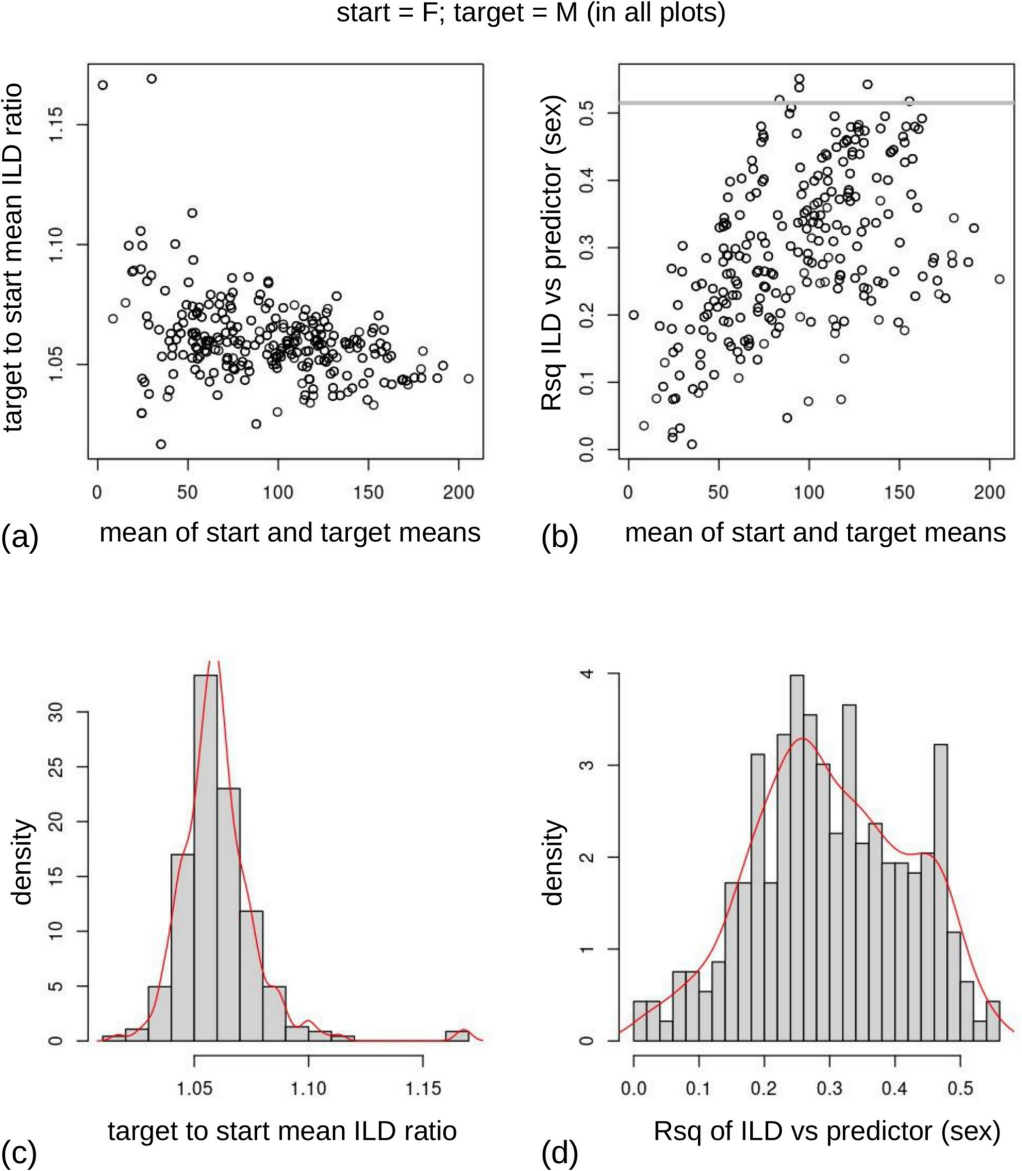
Optional *ILDSR2()* plots, with (**a**-**c**) specific to Procrustes form data (see Discussion for details): (**a**) average male to female ILD ratios vs. ILD grand mean; **b**) ILD-predictor Rsq vs. grand mean of ILD, with a grey line marking the top 2% (highest R²); (**c**) histograms of ILD average male to female ratios and (**d**) ILD-predictor Rsq

Setting the option *plot = TRUE*, four diagrams are shown in the same window, as illustrated in Fig. 5 using the entire Australian sample. All these plots, except the last one (Fig. 5d), are aimed at aiding the interpretation of results in relation to the differences between, by default, the means of the two groups, which are referred to as *start* and *target*. *Start* is used for the mean of group one (first level of the factor, which is F, the females, in our case) and *target* for the mean of the second group (second level of the factor, M, and, thus, males in our example). One could swap the order of the groups, whereas, if a covariate was used instead of a two group factor, *start* and *target* would correspond to its minimum and maximum (i.e., the opposite endpoints of the variation in the covariate). For example, *start* and *target* might be the shortest and highest individual in a sample, if the covariate was body height, or the smallest and largest cranium, if the covariate was a measure of overall cranial size, such as the centroid size of the landmark configuration.

In each of the four diagrams, summary statistics are computed for each ILD. The first plot in Fig. 5a illustrates the ratio of the mean ILD for males to the mean ILD for females in the Australian sample, relative to the overall mean of these two values. For instance, using GLAMA as an example, on the vertical axis there will be the ratio of male to female mean GLAMA (137.5 mm/127.4 mm = 1.08) and on the horizontal one there will be the mean of female and male GLAMA means ((127.4 mm + 137.5 mm)/2 = 132.5 mm). Figure 5b has the same horizontal axis as 5a but shows the Rsq of each ILD (e.g., GLAMA’s Rsq for sex = 0.54) on the vertical one. Therefore, in Fig. 5a, we see that men have always larger ILDs on average (ratios of means > 1), but there is a fair amount of variability. Depending on the specific ILD, their cranial measurements can be up to 17% larger than in females. However, ratios of means of shorter ILDs (left side of the plot) tend to vary much more than larger ones (right side of the plot), with the largest ILDs seemingly converging on a male to female mean ratio of about 1.05 (i.e., on average 5% longer in men). In spite of including the ILDs with the largest male to female ratios (values above 1.1 in the left side of Fig. 5a), and thus with the largest mean differences, Rsq (Fig. 5b) tend to be lower in smaller ILDs, larger for intermediate measurements (~ 90–150 mm) and intermediate for the largest cranial measurements. Figures 5c and d respectively show, using histograms, the density of ILDs in relation to the ratio between group means or the ILD Rsq. For the ratios (Fig. 5c), there is a clear peak indicating that a majority of cranial measurements tend to be 5% larger in men. For Rsq (Fig. 5d), the distribution is left-skewed and peaks slightly below 0.3 (i.e., 30% of size variation accounted for by sex).

As anticipated, these plots are optional and should never be used on raw Cartesian coordinates. All of them, except the Rsq histogram (Fig. 5d), require data to be in a form space, which is simply obtained by Procrustes superimposing the data [54] and then by rescaling the coordinates to (i.e., multiplying for) the original centroid size (Klingenberg, 2016). Using group means in form space can be seen as a computational shortcut, whose results most of the time will be virtually identical to those of calculations done directly on the ILDs. For instance, to compute ratios between mean male and female ILDs, one could simply average each ILD by sex and do the corresponding ratio. Right now, in contrast, the function computes for each sex the mean of the form coordinates and then obtains the mean ILDs from the two form averages. Thus, the superimposition used to compute form may have an effect on results, although it is likely to be relatively inconsequential most of the time. In our case, using again GLAMA as an example, directly averaging this ILD by sex produces a female mean of 127.4 mm and a male mean of 137.5 mm. Using the form space procedure, as currently implemented in *ILDSR2()*, the corresponding means are respectively 127.4 and 137.4 mm. Results are, therefore, identical for females and differ for males by 1/10 of a mm. Yet, the impact might vary from case to case and, for this reason, the plots using mean ILDs from averaged forms should be used carefully. More importantly, they cannot be used when the matrix in *ILDSR2()* contains raw coordinates, since mean ILDs would be derived from averaged coordinates that contain spurious differences due to translational and rotational differences during landmark digitization.

### ILDs variability? Most sexually dimorphic measurements may not be those with largest mean differences

In addition to demonstrating the potential of *ILDSR2()*, the example dataset we analysed revealed further interesting observations. The variability of the most discriminatory ILDs in relation to the specific composition of a randomized subsample was not unexpected, albeit higher than forecast. In considering that the most discriminatory ILDs may change depending on the randomized subsample, and that Rsq can be higher in some top ILDs that were supported by a small number of subsamples (Fig. 2c), indicates that relying only on bootstraps and the magnitude of Rsq can be misleading. Indeed, top ILDs varied across subsamples in number (from one to four) and in terms of the specific pair of landmarks involved. This variability occurred despite the relatively large size of the subsample (comprising 50 individuals per sex) and the partial overlap in the composition of each randomized subsample. Informative osteometric data in forensic application are typically population-specific [25, 38–41]. However, our analysis indicates that even within a population from a well-defined and relatively circumscribed geographic region, such as Western Australia, cranial form can be remarkably variable. Thus, it seems unlikely that specific ILDs, such as GLAMA or NAZYG, will consistently be the most accurate for classifying sex in new samples, even if they are relatively accurate predictors in that population.

Focusing on the most discriminatory ILDs, such as those found at least in 10% of the subsamples (emphasized in violet-yellow in Figs. 1b and 2), our analysis suggested that length measurements in the region between the fronto-nasal area and the zygomatic bone-mastoid process can be particularly effective discriminating skeletal sex. It is well known that the forehead contour (including the glabella), the zygomatic arch and mastoid process are among the most sexually dimorphic regions in adult human crania [21]. In our dataset, these distances were robust to bootstrapping and two (GLAMA and NAZYG) were supported by the majority of random subsamples. Expectedly, the most supported top ILDs generally had the highest Rsq (Fig. 2c), but they were not necessarily those with the largest average between-sex difference (Fig. 5a-b; see previous subsection). In fact, GLAMA and NAZYG were just ~ 8% larger in males than females, but it was evident that the largest group mean difference in the Australian sample can, for some ILDs, be twice as large. Specifically, males were 17% larger than females on average using the distance measurement from frontozygomatic orbitale to frontomalare temporale, and also from porion to mastoidale (in both cases with the landmarks on the same side of the cranium). These are the two ILDs that resemble outliers in Fig. 5a (top left corner). However, the latter figure also shows that the two largest mean differences correspond to some of the smallest ILDs (Fig. 5a). In these ILDs their variance is proportionally larger than in GLAMA and NAZYG, and is likely to some extent inflated by a stronger impact of measurement error on smaller distances [55]. The relatively larger variability of the two small ILDs with the largest average sex difference can be inferred by comparing, within each sex, the coefficient of variation, which is the standard deviation divided by the mean, of the corresponding measurements. The coefficient of variation was 0.11–0.16 for those two ILDs compared to just 0.03–0.04 for GLAMA and NAZYG. Thus, smaller ILDs may have a higher mean difference between sexes, but can be poor predictors to identify females and males because of their large within-sex variability. With more variability, group overlap increases, and thus classification accuracy can be reduced. In contrast, the best supported sex-related ILDs combined moderately large differences with small within group variance, which implied less overlap between measurements of females and males, and therefore more accurate discrimination.

### The Italian sample as an example of external validation

GLAMA and NAZYG showed high accuracy and low sex bias in the cross-validated DA of the Australian sample. However, when the same measurements for the Italian sample were plotted onto the PP contour plot from the Australian data (Fig. 4b), the average sex classification accuracy dropped to approximately 64%, with all misclassified individuals being males; this demonstrates a significant sex bias. It also raises the question of whether such a poor performance mainly originates from the specific choice of ILDs, the sample-specific predictive function, or both factors combined.

The external validation of the results from the Australian dataset exemplified in Fig. 4b is simultaneously assessing the impact of multiple factors on sex classification using the independent sample from Italy. The first factor is the selection of the most supported sexually dimorphic top ILDs in the Australian sample. We have already discussed how, even if GLAMA and NAZYG were the most frequently supported ILDs in this dataset, they failed to reach the 50% bootstrap threshold in more than 1/3 of the randomized Australian subsamples. If there is variability within the training dataset from Australia, indicative of a moderate degree of internal validity, it is reasonable to assume that the same randomization experiment would produce different top ILDs in the Italian sample. A second factor impacting classification accuracy in the Italian sample is the DA function, which was computed using the Australian data, and thus produces PPs specific to that sample. When we instead computed PPs in a DA using GLAMA and NAZYG in the Italian sample, the cross-validated average classification accuracy were 80% with a 16% female bias (i.e., 89% female accuracy vs. 73% for males). This result is relatively close to the 84% of Milella et al. using cranial centroid size in the same Italian sample. However, the sex bias value was just 1% in the classification of Milella et al. [44]. In contrast, with a high associated strong sex bias value, GLAMA and NAZYG appear to not be the most accurate option to classify sex in the Italian sample even when the DA function is specific to this sample. That the poor performance of these measurements, when applied to Italians, originates from both the selected ILDs as well as the specific predictive function, is suggested also by a previous study, which used functions based on a Euro-American sample to classify adult sex in an Italian craniometric dataset [56]. Although the linear measurements employed were different, a strong female bias was observed, with almost all misclassified individuals being males. Furthermore, the authors reported larger glabellar projections and mastoid heights in Euro-Americans compared with Italians, which helps explain why GLAMA may be particularly less accurate for sex prediction in the latter.

The notion that DAs tend to generalize poorly across human geographic samples is well established [31, 57] and could be especially true for size measurements, such as cranial ILDs. The same basic relationship is also known to have the same influence on generalizing the expression of morphoscopic (shape) features used to classify sex [37, 58]. This is evident in our study case, with the contemporary Australian population having on average a significantly larger cranium than the archaeological sample from Italy. This large size difference is probably not only related to geographic separation, but also a consequence of temporal variation, with the Italian population almost a century older than the Australian sample. The relative effect of secular variation and body size is well quantified and it is known that stature has increased significantly in the last few generations especially in the wealthiest nations [59, 60]. Thus, as Italian women living a century ago were smaller than contemporary Australians, they were correctly classified based on cranial size. In contrast, the smaller crania of Italian men in the archaeological sample positioned them roughly halfway between modern Australian women and men in the GLAMA–NAZYG data space (Fig. 4b), leading to two-thirds of them being misclassified as female.

### Sex-related ILDs selection

For the selection of predictors, we only focused on Rsq in relation to groups. However, one might also want to consider whether predictors are highly collinear (i.e., strongly correlated) and thus whether there is redundancy in the data. High collinearity can impact results of DAs and other statistical analyses [61]. The impact may be secondary, in terms of accuracy, when the aim is pure classification. Yet, it might affect the strength of sample dependency in the selected sex-related ILDs. With high collinearity, the most discriminatory ILDs may be unstable, because a small change in sample composition may lead to one or another ILD having the highest correlation with sex. Resampling methods, such as bootstrapping (as done using *HILDR2()*) and randomizing subsamples, facilitates exploration of these types of impacts. If a specific ILD is repeatedly found in most bootstrapped and/or randomized subsamples, then this suggests a degree of robustness and negligible collinearity effect.

In our study, the correlation between GLAMA and NAZYG was high (*r* = 0.82). However, this is expected and thus somewhat inevitable in cranio-facial measurements relative to a general scaling effect with increasing cranial size. For instance, among hundreds of ILDs, the median pairwise correlation in the Australian dataset was 0.51 with a 10th-90th interquartile range of 0.28–0.76. In this respect, computing ILDs from symmetrized form data and discarding identical symmetric ILDs helps to reduce redundancy and collinearity, because the same distances measured on different sides of the cranium are typically very highly correlated. For instance, in our dataset, this approach reduced the number of ILDs by almost 50%, from 465 to 249. Further, GLAMA and NAZYG were robust to resampling, which indicates that, despite collinearity, at least for the specific Australian sample, they are only modestly impacted by the precise composition of samples.

As we demonstrated, exploring bootstrapped Rsq between ILDs from landmark coordinates and a two group factor can be useful. Alternatively, however, users may simply employ *ILDSR2()* to compute all pairwise ILDs, and then if optimal classification is the aim, employ a stepwise cross-validated DA (or another classification method) to select the most discriminatory subset of predictors. It is important to observe that, in applying this approach, there is no exploration of support using resampling methods, and that stepwise DAs are easily misused and/or misinterpreted [62]. In further considering the latter, the following explores the results we would have obtained for the Australian sample. With the Wilk’s Lambda criterion in a stepwise forward variable selection for classification [62], five ILDS are selected, resulting in 92% correct classification by sex in a leave-one out cross-validated DA (0% sex bias; 80% correct classification with a PP ≥ 0.8 for either sex). Of those five predictors, two are the same (i.e., GLAMA and NAZYG) identified using bootstraps in randomized subsamples. Also, the improvement in sex classification accuracy appears minimal using the stepwise DA approach and it comes at the cost of adding three more variables to the model. Furthermore, unlike GLAMA and NAZYG, none of the three ‘extra’ predictors found in the stepwise DA (not shown) appeared among the first five sex-related top ILDs in Fig. 2a-b, which suggests that they are not robust to sample composition. Nonetheless, the stepwise DA is a potentially valid option, with its main advantage being faster computation.

### PP contour scatterplot

With just two predictors, as demonstrated in our study case, plotting new observations (Italian) in the data space of a training sample (Australian), and using a contour plot to visualize predicted PPs, is an effective and relatively simple approach to infer the classification of a new dataset. If this is done with appropriate caution, one could even use this type of graphical analysis in the field for a preliminary assessment of sex after measuring the selected ILDs with a sliding caliper. For further accuracy, users should carefully consider instrumental and inter-operator differences [63, 64]. Ideally, for field use, the PP contour plot should be redrawn using linear measurements acquired with a caliper following a standardized easy-to-follow protocol. Adding a square grid could also help precisely map new individuals in the data plot. Nevertheless, results will be a first approximation for classification of skeletal sex. As is customary in forensics, and clearly exemplified by poor performance when applied to the Italian sample, the PP contour scatterplot is population specific. Different ILDs may perform more accurately than GLAMA and NAZYG in a different population and certainly, even if the same ILDs were used, PPs should be computed with a DA specific to that population. Thus, users of a PP contour scatterplot should be aware of and acknowledge its limitations, and also avoid over-interpretations. For instance, even in an accurate population-specific and validated PP contour scatterplot, individuals falling near the 0.5 PP probability boundary should be clearly flagged as very low confidence cases, if classified as females or males. More generally, together with the identification of sex, one should report the PP of each individual or, as in Fig. 4, specify in which range of PPs an individual falls (e.g., between 0.5 and 0.6 PP of being a male, and the like).

With more than two ILDs as sex predictors, one cannot make a PP contour scatterplot in the space of the observed measurements (i.e., one ILD on X, the other on Y with contour lines for the DA PPs estimated in a training sample using those two ILDs). A related type of plot can still be used for visualizing group differences. The latter requires summarizing variation with a between group PCA [65, 66] and using the between group axis (with a two level factor, such as sex, there is only one) vs. the PC1 of the residual, non-group related, variance to plot the individuals. The PP contour lines will be the interpolated values based on the DA PPs. Such a plot, however, loses the appeal of simplicity and the potential application in the field of a graphical summary based on just two real measurements, as in Fig. 4, because it requires the projection of ILDs on vectors of coefficients to reduce dimensionality. Nonetheless, the summary visualization of differences one obtains can be effective and useful.

## Conclusions

Traditional morphometric analysis of ILDs remains useful in many fields and has important practical application in forensics. We have presented a resampling approach based on applying a new R function that facilitates the computation of hundreds of ILDs from Cartesian coordinates of anatomical landmarks and selection of a subset that has a high and robust association with a two-group factor, such as sex. We have also demonstrated how ILDs selected using this approach are accurate predictors of skeletal sex based on cranial size. In this respect, although predictive accuracy was cross-validated, we can only claim its internal sample-specific validity. The poor performance when results were applied to a different sample (the Italian dataset) was expected relative to temporal and geographic variance of the Australian sample. Furthermore, with different operators and methods used to obtain the landmark coordinates, population differences may be inflated and inaccuracy increased when standards derived from the Australian sample were applied to the Italian one. We used the Italian dataset for purely didactic reasons, as it was available and had many landmarks in common with the Australian sample. It would be interesting in the future to explore what happens if the Australian PP contour plot was used to estimate sex in a new sample from the same, or a different region, of Australia. Further, although predictive models must be population specific and, therefore, different PP contour plots must be computed for different geographical regions and ancestry groups, it will be interesting to apply the same approach we used to sex-related ILD selection. This will facilitate exploration of differences and consistencies across populations in the specific cranial measurements that are robustly associated with sexual dimorphism. By expanding the analysis in this direction, we might achieve insight that is not only useful for forensic application, but more broadly provide interesting information on craniofacial sexual dimorphism.

## References

[1] Garvin HM, Klales AR (2020) Adult skeletal sex estimation and global standardization. Forensic Science and Humanitarian Action: Interacting with the Dead and the Living. In: Parra RC, Zapico SC, Ubelaker DH (eds). John Wiley \& Sons, Ltd, Chichester, UK, pp 199–209

[2] Dunsworth HM (2020) Expanding the evolutionary explanations for sex differences in the human skeleton. Evol Anthropol Issues News Rev 29:108–116. 10.1002/evan.21834 10.1002/evan.21834 32359124

[3] Waxenbaum EB, Feiler ME (2021) Influence of climatic stress on nonmetric sexually dimorphic features of the skull and pelvis. Am J Hum Biol 33:e23559. 10.1002/ajhb.23559 33377211 10.1002/ajhb.23559

[4] Lopes Cardoso I, Moreira MT, Dupuis C et al (2024) Amelogenin-based molecular methods for sexual dimorphism identification: protocol of a scoping review. Forensic Sciences 4:499–507. 10.3390/forensicsci4040033

[5] Maulani C, Auerkari EI (2020) Molecular analysis for sex determination in forensic dentistry: a systematic review. Egypt J Forensic Sci 10:36. 10.1186/s41935-020-00210-6

[6] Mikšík I, Morvan M, Brůžek J (2023) Peptide analysis of tooth enamel – a sex estimation tool for archaeological, anthropological, or forensic research. J Sep Sci 46:2300183. 10.1002/jssc.202300183 10.1002/jssc.202300183 37232204

[7] Morikawa T, Yamamoto Y, Miyaishi S (2011) A new method for sex determination based on detection of SRY, STS and amelogenin gene regions with simultaneous amplification of their homologous sequences by a multiplex PCR. Acta Med Okayama 65:113–122 21519369 10.18926/AMO/45270

[8] Walker PL (2008) Sexing skulls using discriminant function analysis of visually assessed traits. Am J Phys Anthropol 136:39–50. 10.1002/ajpa.20776 18324631 10.1002/ajpa.20776

[9] Walker PL (2005) Greater sciatic notch morphology: sex, age, and population differences. Am J Phys Anthropol 127:385–391. 10.1002/ajpa.10422 15693026 10.1002/ajpa.10422

[10] Baumgarten SE, Kenyon-Flatt B (2020) Chap. 11 - Metric methods for estimating sex utilizing the pelvis. In: Klales AR (ed) Sex Estimation of the Human Skeleton. Academic Press, pp 171–184

[11] Franklin D, Cardini A, Flavel A et al (2013) Concordance of traditional osteometric and volume-rendered MSCT interlandmark cranial measurements. Int J Legal Med. 10.1007/s00414-012-0772-9 23052442 10.1007/s00414-012-0772-9

[12] Murail P, Br\uužek J, Houët F, Cunha E (2005) DSP: a tool for probabilistic sex diagnosis using worldwide variability in hip-bone measurements. Bulletins et mémoires de la Société d’Anthropologie de Paris BMSAP 17:167–176

[13] Bytheway JA, Ross AH (2010) A geometric morphometric approach to sex determination of the human adult os coxa. J Forensic Sci 55:859–864. 10.1111/j.1556-4029.2010.01374.x 20384930 10.1111/j.1556-4029.2010.01374.x

[14] Franklin D, Freedman L, Milne N, Oxnard C (2006) A geometric morphometric study of sexual dimorphism in the crania of Indigenous Southern Africans. S Afr J Sci 102:229–238

[15] Franklin D, Cardini A, Flavel A, Kuliukas A (2012) The application of traditional and geometric morphometric analyses for forensic quantification of sexual dimorphism: preliminary investigations in a Western Australian population. Int J Legal Med 126:549–558. 10.1007/s00414-012-0684-8 22399102 10.1007/s00414-012-0684-8

[16] Del Bove A, Profico A, Riga A et al (2020) A geometric morphometric approach to the study of sexual dimorphism in the modern human frontal bone. Am J Phys Anthropol 173:643–654. 10.1002/ajpa.24154 33025582 10.1002/ajpa.24154

[17] Del Bove A, Menéndez L, Manzi G et al (2023) Mapping sexual dimorphism signal in the human cranium. Sci Rep 13:16847. 10.1038/s41598-023-43007-y 37803023 10.1038/s41598-023-43007-y PMC10558540

[18] Marcus LF (1990) Traditional morphometrics. In: Proceedings of the Michigan morphometrics workshop. Special Publication, pp 77–122

[19] Neff NA, Marcus LF (1980) A survey of multivariate methods for systematics. American Museum of Natural History, New York

[20] Krishan K, Chatterjee PM, Kanchan T et al (2016) A review of sex estimation techniques during examination of skeletal remains in forensic anthropology casework. Forensic Sci Int 261:165.e1-165.e8. 10.1016/j.forsciint.2016.02.007 26926105 10.1016/j.forsciint.2016.02.007

[21] Rösing FW, Graw M, Marré B et al (2007) Recommendations for the forensic diagnosis of sex and age from skeletons. HOMO 58:75–89. 10.1016/j.jchb.2005.07.002 17306261 10.1016/j.jchb.2005.07.002

[22] Wang X, Liu G, Wu Q et al (2024) Sex estimation techniques based on skulls in forensic anthropology: a scoping review. PLOS ONE 19:e0311762. 10.1371/journal.pone.0311762 39652615 10.1371/journal.pone.0311762 PMC11627412

[23] Franklin D, Marks MK (2022) The professional practice of forensic anthropology: contemporary developments and cross-disciplinary applications. WIREs Forensic Science 4:e1442. 10.1002/wfs2.1442

[24] Elliott M, Collard M (2009) FORDISC and the determination of ancestry from cranial measurements. Biol Lett 5:849–852. 10.1098/rsbl.2009.0462 19586965 10.1098/rsbl.2009.0462 PMC2827999

[25] Komar DA, Buikstra JE (2008) Forensic anthropology: contemporary theory and practice. Oxford University Press

[26] Franklin D (2022) Estimation of Skeletal Sex. In: Eldridge H, Lewis S, Lothridge K (eds) Encyclopedia of Forensic Sciences, Third Edition. Elsevier, pp 292–303

[27] Küchler EC, Kirschneck C, Marañón-Vásquez GA et al (2024) Mandibular and dental measurements for sex determination using machine learning. Sci Rep 14:9587. 10.1038/s41598-024-59556-9 38671054 10.1038/s41598-024-59556-9 PMC11053013

[28] Holland TD (1986) Sex determination of fragmentary crania by analysis of the cranial base. Am J Phys Anthropol 70:203–208. 10.1002/ajpa.1330700207 3740247 10.1002/ajpa.1330700207

[29] Spradley MK, Jantz RL (2011) Sex estimation in forensic anthropology: skull versus postcranial elements. J Forensic Sci 56:289–296. 10.1111/j.1556-4029.2010.01635.x 21210801 10.1111/j.1556-4029.2010.01635.x

[30] France DL (1998) Observational and metric analysis of sex in the skeleton. Forensic osteology: advances in the identification of human remains 163–186

[31] Franklin D, Blau S (2020) Chap. 1.3 - Physical and virtual sources of biological data in forensic anthropology: Considerations relative to practitioner and/or judicial requirements. In: Obertová Z, Stewart A, Cattaneo C (eds) Statistics and Probability in Forensic Anthropology. Academic Press, pp 17–45

[32] Franklin D, Flavel A (2019) Population specificity in the estimation of skeletal age and sex: case studies using a Western Australian population. Aust J Forensic Sci 51:S188–S192. 10.1080/00450618.2019.1569722

[33] Franklin D, Cardini A, Flavel A, Marks MK (2014) Morphometric analysis of pelvic sexual dimorphism in a contemporary Western Australian population. Int J Legal Med 128:861–872. 10.1007/s00414-014-0999-8 24789357 10.1007/s00414-014-0999-8

[34] Franklin D (2023) Estimation of skeletal sex. In: Houck MM (ed) Encyclopedia of forensic Sciences, third edition (Third edition), third edition. Elsevier, Oxford, pp 292–303

[35] Ubelaker DH, DeGaglia CM (2017) Population variation in skeletal sexual dimorphism. Forensic Sci Int 278:407.e1-407.e7. 10.1016/j.forsciint.2017.06.012 28698063 10.1016/j.forsciint.2017.06.012

[36] Swift L, Obertova Zuzana, Flavel Ambika et al (2023) Estimation of sex from cranial measurements in an Australian population. Aust J Forensic Sci 55:755–770. 10.1080/00450618.2022.2081358

[37] Lye R, Obertová Z, Bachtiar NA, Franklin D (2024) Validating the use of clinical MSCT scans for cranial nonmetric sex estimation in a contemporary Indonesian population. Int J Legal Med 138:1559–1571. 10.1007/s00414-024-03176-5 38300302 10.1007/s00414-024-03176-5 PMC11164787

[38] Franklin D, Freedman L, Milne N (2005) Sexual dimorphism and discriminant function sexing in indigenous South African crania. HOMO 55:213–228. 10.1016/j.jchb.2004.08.001 15803767 10.1016/j.jchb.2004.08.001

[39] Frutos LR (2005) Metric determination of sex from the humerus in a Guatemalan forensic sample. Forensic Sci Int 147:153–157. 10.1016/j.forsciint.2004.09.077 15567620 10.1016/j.forsciint.2004.09.077

[40] Kranioti EF, Michalodimitrakis M (2009) Sexual dimorphism of the humerus in contemporary Cretans—a population-specific study and a review of the literature. J Forensic Sci 54:996–1000. 10.1111/j.1556-4029.2009.01103.x 19627412 10.1111/j.1556-4029.2009.01103.x

[41] Steyn HS, Ellis SM (2009) Estimating an effect size in one-way multivariate analysis of variance (MANOVA). Multivar Behav Res 44:106–129. 10.1080/00273170802620238 10.1080/00273170802620238 26795108

[42] R Core Team (2025) R: A Language and Environment for Statistical Computing. R Foundation for Statistical Computing, Vienna, Austria

[43] Schlager S (2017) Morpho and Rvcg – Shape Analysis in R. In: Zheng G, Li S, Szekely G (eds) Statistical Shape and Deformation Analysis. Academic Press, pp 217–256

[44] Milella M, Franklin D, Belcastro MG, Cardini A (2021) Sexual differences in human cranial morphology: is one sex more variable or one region more dimorphic? Anat Rec 304:2789–2810. 10.1002/ar.24626 10.1002/ar.24626 33773067

[45] Klingenberg CP (2016) Size, shape, and form: concepts of allometry in geometric morphometrics. Dev Genes Evol 226:113–137. 10.1007/s00427-016-0539-2 27038023 10.1007/s00427-016-0539-2 PMC4896994

[46] Klingenberg CP, Barluenga M, Meyer A (2002) Shape analysis of symmetric structures: quantifying variation among individuals and asymmetry. Evolution 56:1909–1920. 10.1111/j.0014-3820.2002.tb00117.x 12449478 10.1111/j.0014-3820.2002.tb00117.x

[47] Claude J (2008) Morphometrics with R. Springer Science & Business Media

[48] Hammer O, Harper D, Ryan P (2001) PAST: paleontological statistics software package for education and data analysis. Paleontol Electron 4(1):1–9

[49] Albrecht G (1992) Assessing the affinities of fossils using canonical variates and generalized distances. Hum Evol 7:49–69

[50] Nychka D, Furrer R, Paige J, Sain S (2021) fields: Tools for spatial data

[51] Rohlf FJ (2021) Why clusters and other patterns can seem to be found in analyses of high-dimensional data. Evol Biol 48:1–16. 10.1007/s11692-020-09518-6

[52] Cardini A (2014) Missing the third dimension in geometric morphometrics: how to assess if 2D images really are a good proxy for 3D structures? Hystrix Ital J Mammal 25:73–81

[53] Roth V (1993) On three-dimensional morphometrics, and on the identification of landmark points. Contributions to Morphometrics Museo Nacional de Ciencias Naturales, Madrid, pp 41–61

[54] Rohlf FJ, Slice D (1990) Extensions of the procrustes method for the optimal superimposition of landmarks. Syst Zool 39:40–59. 10.2307/2992207

[55] Polly PD (1998) Variability in mammalian dentitions: size-related bias in the coefficient of variation. Biol J Linn Soc 64:83–99

[56] Manthey L, Jantz RL, Vitale A, Cattaneo C (2018) Population specific data improves Fordisc®’s performance in Italians. Forensic Sci Int 292:263.e1-263.e7. 10.1016/j.forsciint.2018.09.023 30342922 10.1016/j.forsciint.2018.09.023

[57] Swift L, Obertova Z, Franklin D (2024) Demonstrating the empirical effect of population specificity of anthropological standards in a contemporary Australian population. Int J Legal Med 138:537–545. 10.1007/s00414-023-03031-z 37269396 10.1007/s00414-023-03031-z PMC10861720

[58] Rojas González N, Obertová Z, Franklin D (2024) Validation and recalibration of sex estimation methods using pubic nonmetric traits for the Chilean population. Int J Legal Med 138:2071–2080. 10.1007/s00414-024-03233-z 38613625 10.1007/s00414-024-03233-z PMC11306301

[59] Cavelaars AEJM, Kunst AE, Geurts JJM et al (2000) Persistent variations in average height between countries and between socio-economic groups: an overview of 10 European countries. Ann Hum Biol 27:407–421. 10.1080/03014460050044883 10942348 10.1080/03014460050044883

[60] Perkins JM, Subramanian SV, Davey Smith G, Özaltin E (2016) Adult height, nutrition, and population health. Nutr Rev 74:149–165. 10.1093/nutrit/nuv105 26928678 10.1093/nutrit/nuv105 PMC4892290

[61] Hair JF, Babin BJ (2018) Multivariate Data Analysis. Cengage

[62] Huberty CJ (1984) Issues in the use and interpretation of discriminant analysis. Psychol Bull 95:156–171. 10.1037/0033-2909.95.1.156

[63] Arnqvist G, Martensson T (1998) Measurement error in geometric morphometrics: empirical strategies to assess and reduce its impact on measures of shape. Acta Zool Acad Sci Hung 44:73–96

[64] Fruciano C (2016) Measurement error in geometric morphometrics. Dev Genes Evol 226:139–158. 10.1007/s00427-016-0537-4 27038025 10.1007/s00427-016-0537-4

[65] Cardini A, Polly PD (2020) Cross-validated between group PCA scatterplots: a solution to spurious group separation? Evol Biol. 10.1007/s11692-020-09494-x

[66] Yendle PW, MacFie HJH (1989) Discriminant principal components analysis. J Chemom 3:589–600. 10.1002/cem.1180030407

[67] Weihs C, Ligges U, Luebke K, Raabe N (2005) klaR Analyzing German Business Cycles. In: Baier D, Decker R, Schmidt-Thieme L (eds) Data Analysis and Decision Support. Springer-Verlag, Berlin, pp 335–343

